# Short-Term Waterlogging in Citrus Rootstocks

**DOI:** 10.3390/plants10122772

**Published:** 2021-12-15

**Authors:** Margarita Pérez-Jiménez, Olaya Pérez-Tornero

**Affiliations:** Equipo de Mejora Genética de Cítricos, Instituto Murciano de Investigación y Desarrollo Agrario y Alimentario (IMIDA), 30150 Murcia, Spain; olalla.perez@carm.es

**Keywords:** Cleopatra mandarin, Forner Alcaide, Alemow, *Citrus macrophylla*, flooding, water stress

## Abstract

Changes in climate are provoking flooding events that cause waterlogging in the fields. Citrus are mainly cultivated in areas with a high susceptibility to climate change. Therefore, it is vital to explore their responses to these events to anticipate future challenges by means of genetic improvement of the commercial rootstocks. In this experiment, three popular commercial rootstocks, namely ‘Cleopatra’ (*C. reshni* Hort. Ex Tanaka), *C. macrophylla*, and ‘Forner Alcaide no. 5′ (*Citrus reshni* Hort. Ex Tanaka × *Poncirus trifoliata*), were evaluated after being submitted to short-term waterlogging and a period of recovery of 7 days in each case. Photosynthesis rate and stomatal conductance decreased in ‘Cleopatra’, while in the other two genotypes they were maintained (*C. macrophylla*) or restored after recovery (‘Forner Alcaide no. 5′’). Relative water content and chlorophylls also decreased in ‘Cleopatra’. This indicates a deeper effect of flooding in ‘Cleopatra’, which suffered changes during flooding that were also sustained during the recovery phase. This did not occur in the other two rootstocks, since they showed signs of recovery for those parameters that decreased during waterlogging.

## 1. Introduction

The climate is changing, and this is causing an increase in temperatures and alterations in water cycles, as reflected by the increasing severity of droughts and by aberrant heavy precipitations that can lead to waterlogging and flooding [1]. This is of particular importance in areas already prone to flooding, such as the Mediterranean basin, where flooding events are increasing. Citrus plants are of special relevance in this area and, although there are several cultivars and rootstocks on the market that are supposedly adapted to a wide range of edaphoclimatic conditions, there is an urgent need to study these genotypes to know if they will cope with the circumstances [2].

Flooding generates hypoxia, which triggers a wide range of metabolic, hormonal, developmental, and physiological processes in plants. Waterlogging decreases Na while increasing K in both leaf and root [3]. Photosynthetic activity decreases mainly due to stomatal closure, a decline in the activity of the Rubisco enzyme, and the destruction of chlorophyll [4]. This leads to the occurrence of desiccation symptoms and a delay in growth. Root growth and transporter-driven ion uptake are also inhibited due to faulty root respiration. This causes an energy shortage and, therefore, a reduced ion uptake and less energy for root growth. Hypoxia is of special relevance to citrus plants because of the lack of a specific adaptation, such as aerenchyma formation or lenticel hypertrophy [5]. However, the capacity to endure waterlogging conditions depends on the genotype, and a wide range of responses to waterlogging has been found in different citrus genotypes [6]. For example, ‘Cleopatra’ has been described as very sensitive to flooding [5], while an excellent tolerance to flooding has been observed in ‘Forner Alcaide no. 5′ [7]. Such information is especially important when it comes to plant citrus breeding, since an accurate evaluation of the physiological features of the germplasm available for breeding will provide breeders with better tools to obtain new rootstocks adapted to the conditions associated with climate change.

Most of the waterlogging studies concerning citrus refer to more than 30 days of flooding [5,8,9,10,11,12]. However, such long periods do not reflect the natural conditions of the Mediterranean basin, where waterlogging may last a week at most. In the above reports, the authors studied ‘Citrumelo’ (*Citrus paradisi* L. Macf. *Poncirus trifoliata* L. Raf.), ‘Carrizo’ (*Carrizo citrange*), and ‘Cleopatra’ *C. reshni* Hort. Ex Tanaka) rootstocks, but not other important rootstocks, such as *C. macrophylla*, a commonly used rootstock that performs well with *C. lemon*, a commercial crop of paramount importance in Spain [13].

In this experiment, three popular commercial rootstocks, namely ‘Cleopatra’ (*C. reshni* Hort. Ex Tanaka), *C. macrophylla*, and ‘Forner Alcaide no. 5′ (*Citrus reshni* Hort. Ex Tanaka x *Poncirus trifoliata*), were evaluated to figure out their capacity to resist floods and to recover after them. Plants were submitted to flooding and growth, water, gas exchange conditions, and ion concentration were measured.

## 2. Results

**Figure 1:**
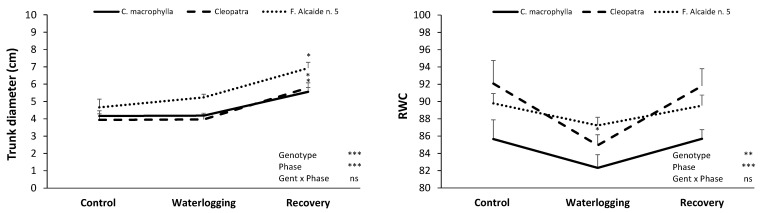
Effects of waterlogging on trunk growth and relative water content (RWC) of *Citrus macrophylla*, ‘Cleopatra’, and ‘Forner Alcaide no. 5′ citrus rootstocks after control, waterlogging, and recovery phases. Values are means ± SE. * denotes significant differences (*p* < 0.05) between plants of the same rootstock after waterlogging and recovery phases and control. (ns *p* ≥ 0.05; * *p* < 0.05; ** *p* < 0.01; *** *p* < 0.001).

Plants of the three studied rootstocks exhibited both anatomical and physiological changes during the experiment. The ANOVA showed significant differences in trunk diameter and RWC according to the genotype and the phase. Trunk diameter did not exhibit any change until the recovery phase in any of the studied rootstocks, preserving its size during waterlogging in all of the three genotypes (Figure 1). On the other hand, RWC decreased after waterlogging in ‘Cleopatra’, while no differences were found in the rest of the genotypes (Figure 1). No differences with regard to control were found after the recovery phase in any of the genotypes.

**Figure 2:**
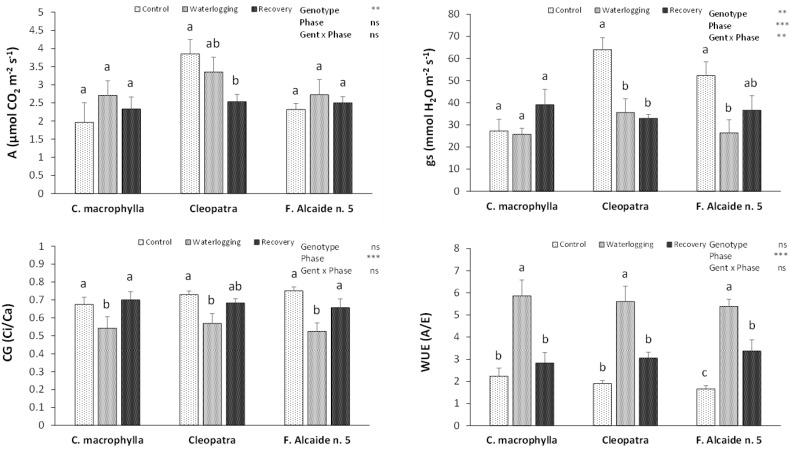
Effects of waterlogging on photosynthesis rate (A), stomatal conductance (gs), carbon gain (Ci/Ca), and water use efficiency (WUE) of *Citrus macrophylla*, ‘Cleopatra’, and ‘Forner Alcaide no. 5′ citrus rootstocks after control, waterlogging, and recovery phases. Values are means ± SE. Different letters denote significant differences (*p* < 0.05) between phases in the same rootstock. (ns *p* ≥ 0.05; ** *p* < 0.01; *** *p* < 0.001).

In terms of gas exchange, although significant differences in the photosynthesis rate were not observed for the phase or the genotype x phase interaction, differences were significant between genotypes; a decrease in the photosynthesis rate was detected in ‘Cleopatra’ after the recovery phase, while no significant changes were found after waterlogging with regard to control and recovery (Figure 2). However, similar photosynthesis rates were found in all of the phases in ‘Forner Alcaide no. 5′ and *C. macrophylla*. Stomatal conductance was significantly affected by genotype, phase, and the interaction of both of them. This parameter dropped after waterlogging and remained low after recovery in ‘Cleopatra’ (Figure 2). In ‘Forner Alcaide no. 5′, a decrease in stomatal conductance after waterlogging was observed; however, conductance proved not to be significantly different between control and waterlogging conditions after the recovery phase. No differences in stomatal conductance between phases were found in *C. macrophylla*. Carbon gain decreased significantly according to the phase in all of the rootstocks after waterlogging (Figure 2), and was restored after recovery in *C. macrophylla* and ‘Forner Alcaide no. 5′. After recovery, no significant differences were found in carbon gain with waterlogging and control in ‘Cleopatra’. Water use efficiency was also significantly affected by the phase, increasing after waterlogging and returning to control levels after recovery in *C. macrophylla* and ‘Cleopatra’ (Figure 2). ‘Forner Alcaide no. 5′ also showed an increased WUE after waterlogging; although WUE decreased again after recovery, the levels at this phase were significantly higher than in control conditions.

**Figure 3:**
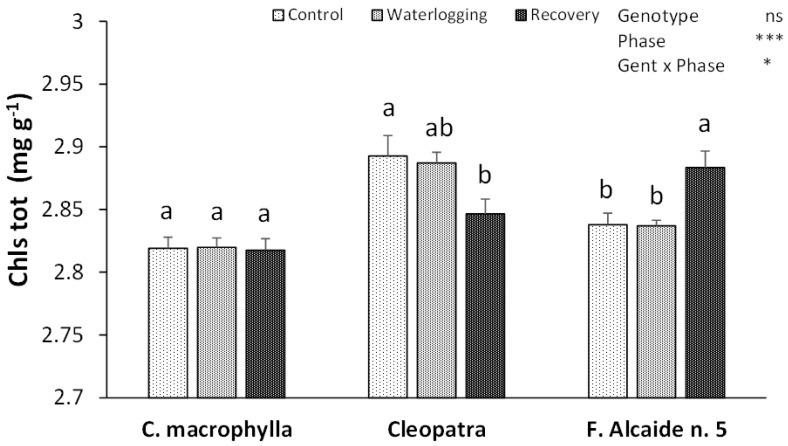
Effects of waterlogging on total chlorophyll content (Chls tot) in leaves of *Citrus macrophylla*, ‘Cleopatra’, and ‘Forner Alcaide no. 5′ citrus rootstocks after control, waterlogging, and recovery phases. Values are means ± SE. Different letters denote significant differences (*p* < 0.05) between phases in the same rootstock. (ns *p* ≥ 0.05; * *p* < 0.05; *** *p* < 0.001).

Chlorophylls did not show any change in *C. macrophylla* during the experiment (Figure 3). However, chlorophylls were affected in ‘Cleopatra’ and ‘Forner Alcaide no. 5′. In ‘Cleopatra’, a lower level of total chlorophylls was detected after the recovery phase than after the application of control conditions. The opposite was found in ‘Forner Alcaide no. 5′, where the level of total chlorophylls detected after recovery was higher than in the previous phases of the experiment.

Concerning ion content, ‘Cleopatra’ did not exhibit any change in ion concentration during the experiment (Table 1). This is in contrast to the variations found in ‘Forner Alcaide no. 5′ and *C. macrophylla*. The response to waterlogging in the ion concentrations of both genotypes was equal in Fe, B, Ca, Cu, and Zn (Table 1). In the case of Fe, Ca, Cu, and Zn, levels were maintained after waterlogging and dropped after recovery with regard to control levels. In ‘Forner Alcaide no. 5′ and *C. macrophylla*, B levels were augmented after waterlogging and were restored after recovery. No changes were found in Mg and Na in ‘Forner Alcaide no. 5′ and in P in *C. macrophylla*, while a decrease after recovery was found in Mn and Na in *C. macrophylla* and in P in ‘Forner Alcaide no. 5′ (Table 1). Finally, *C. macrophylla* exhibited a rise in Mg after recovery, while Mn increased after waterlogging to be restored after recovery in ‘Forner Alcaide no. 5′ (Table 1).

## 3. Discussion

Short-term waterlogging is an adequate representation of typical floods in the Mediterranean regions, where long drought periods combined with torrential rainfall episodes and convective events [14] are common. Floods can damage citrus orchards, creating a need for rootstocks adapted to climate change conditions. Bearing that in mind, knowing the effects on the most important citrus rootstocks and their response to this is essential for using them for culture and as a source of germplasm for breeding. In this experiment, three common rootstocks were tested against waterlogging and a recovery period with no water was applied to learn about, and differentiate between, immediate and delayed effects, and also between transitory and long-term effects. When a stress signal is detected in the cell, a tentative molecular cascade may be activated to overcome transitional stress [3], triggering a fast response from the plant. If the challenge remains, these processes are able to reprogram gene expression to improve survival [15], exhibiting delayed responses. Thus, some changes in plants can be observed after waterlogging, and others after the recovery phase.

In citrus, trunk diameter is very sensitive to water status [16]. In this experiment, plants ceased growing after waterlogging and showed an increase in their diameter after recovery. To avoid desiccation after flooding, plants reacted by increasing their WUE, which involves stopping and slowing down many physiological processes. Similarly, carbon gain is reduced, which cuts down carbon fixation in the leaf. In citrus, reduction in growth has previously been related to impaired photosynthetic activity in waterlogging conditions [11]. However, this link has not been found in this experiment, where variations in photosynthesis cannot be related to differences in stem growth. All of the rootstocks had the same behavior in trunk growth, which is in contrast to the absence of changes in the photosynthesis rate in *C. macrophylla* and ‘Forner Alcaide no. 5′, and to its decrease in ‘Cleopatra’ after recovery, when the trunk started to grow again. This would imply a direct action of the water regimen in fast responses such as WUE and CG, which would be influencing stem growth.

Waterlogging induces desiccation in sensitive genotypes, caused by a reduced ability to take up water [17]. This effect was seen in the present experiment in ‘Cleopatra’, which showed a decreased RWC in the waterlogging phase. These results would indicate a water deficit due to the reduced hydraulic conductance in roots, which leads to stomatal closure [10]. However, the reduction in stomatal conductance seen in plants of ‘Forner Alcaide no. 5′ was not accompanied by changes in RWC, showing the adaptive role of stomatal closure in counteracting leaf dehydration, something that was detected in *Carrizo citrange* in previous studies [10]. As could be seen, one of the first responses to flooding is stomatal closure [18], and with the stomatal closure comes a reduction in gas exchange, which is followed by a fall in transpiration and an increase in the WUE in an attempt to avoid dehydration. This phenomenon has been observed in previous waterlogging studies in citrus [8,10]. Citrus stomata are reported to delay their response until day 8–9 of flooding [6]; however, in our study, this occurred earlier in ‘Cleopatra’ and ‘Forner Alcaide no. 5′. In ‘Cleopatra’, stomata might close as a response to leaf water deficit. On the contrary, in the case of ‘Forner Alcaide no. 5′, since RWC was preserved, changes in gs could have been induced by an abscisic acid hormonal signal transmitted from the roots to the shoots or by the accumulation of this hormone in the leaves, which is involved in plant resistance to flooding [4]. In fact, after recovery gs increased to previous control levels, showing a better behavior in flooding once again.

However, through its effect on gas exchange, stomatal closure also influences photosynthesis rate by restricting the access of CO_2_, as was observed in this experiment in ‘Cleopatra’. ‘Forner Alcaide no. 5′ was able to maintain the photosynthesis rate despite the drop in stomatal conductance, probably due to the activation of Rubisco as a result of hypoxia [19]. Plants show different adaptations that can make them improve their resistance to water stress. Thus, in the present study, *C. macrophylla* and ‘Forner Alcaide no. 5′ were able to preserve their photosynthesis rate during waterlogging, and *C. macrophylla* also maintained its water conditions without any modification of its stomatal conductance. This detrimental effect on the photosynthetic system of ‘Cleopatra’ points towards the vulnerability of this rootstock to short-term waterlogging conditions, as already reported by other authors, who mentioned that ‘Cleopatra’ plants reflect stress symptoms earlier than other genotypes under long-term flooding conditions [9]. The downward rate of photosynthesis in ‘Cleopatra’ seems to be linked not only to the stomatal closure, but also to other side effects of waterlogging, such as chlorophyll degradation, as has been reported in ‘Sour Orange’ (*Citrus aurantium*) [20].

In like manner, nutrients play a major role in the responses detected in waterlogging conditions, due to photosynthesis’s dependence on chlorophyll, or ATP synthesis impairment, which disturbs plant metabolism. However, a direct connection between ion variations and plant status has not been established in this experiment. As previously reported [6], waterlogging tended to maintain nutrient concentration in the case of ‘Cleopatra’ or to decrease it in the case of the rest of the rootstocks. Throughout this experiment, different responses have been found in ‘Cleopatra’, *C. macrophylla* and ‘Forner Alcaide no. 5′, which have probably determined the final water status of each plant due to waterlogging. Previous studies have linked the lack of differences in micronutrients under waterlogging conditions when compared to controls with a higher capacity to endure waterlogging [6]. Nevertheless, the nutrient composition of these three rootstocks has proved that changes in nutrients do not mean a higher sensitivity to waterlogging. In fact, ‘Cleopatra’, the most sensitive genotype in this experiment in terms of plant water status after waterlogging and recovery, was the only genotype where nutrients did not show any change in their amounts.

## 4. Materials and Methods

### 4.1. Plant Material and Experimental Conditions

Plant material was obtained from 1-year-old *C. macrophylla*, ‘Cleopatra’ (*C. reshni* Hort. Ex Tanaka), and ‘Forner Alcaide no. 5′ (*C. reshni* Hort. Ex Tanaka x *Poncirus trifoliata*) citrus rootstocks. Plants were grown in 5 l black containers filled with a commercial mixture of peat (Pelemix, Spain). They were watered daily with a minimum of 35% drainage with nutrient solution until the start of the experiment. Plants were left in the climate chamber for a 30-day adaptation period. The experiment was carried out in a climate chamber with fully controlled environmental conditions: 16 h photoperiod, 25 °C, 60% relative humidity, and photosynthetically active radiation (PAR) of 100 μmol m^−2^ s^−1^. After this period, plants were submitted to waterlogging, achieved by submerging the pots in plastic water tanks (50 l) and maintaining the water level 2 cm above the soil surface. After 7 days, trees were removed from water and drained so that they might recover for 7 additional days.

### 4.2. Plant Growth and Water Status

Vegetative growth was evaluated by measuring the trunk diameter at 10 cm from the substrate using an electronic digital caliper.

Relative Water Content (RWC) was determined using the equation RWC = 100[(FW − DW)/(TW − DW)], where FW is the fresh weight, TW is the turgid weight, and DW is the dry weight of leaf discs. To obtain full turgor, leaf discs were placed in darkness for 24 h in vials containing water to allow for a complete rehydration. DW was determined after drying the leaf discs at 65 °C for 72 h.

### 4.3. Gas Exchange

Net CO_2_ assimilation (A), transpiration rate (E), stomatal conductance (gs), and intercellular CO_2_ concentration (Ci) were measured in the second-youngest fully expanded leaf of each plant, using a portable photosynthesis measurement system (LI-6400, Li-Cor, Lincoln, NE, USA) equipped with a broadleaf chamber. All measurements were taken at an external ambient CO_2_ concentration (Ca) of 400 μmol mol^−1^ CO_2_, a leaf temperature of 25 °C, 60% relative humidity, and a PPFD of 100 μmol m^–2^ s ^–1^. Carbon gain (CG) (Ci/Ca) and water use efficiency (WUE) (A/E) were calculated.

### 4.4. Chlorophylls Determination

Chlorophyll content was estimated following the procedure described by [21], in 20 mg samples of ground material that had been kept at 4 °C in the dark for 48 h with N,N-dimethylformamide. The absorbance of each extract was recorded at 664.5 and 647 nm, and total chlorophyll concentrations (mg kg^−1^ DW) were calculated with the following equation: 17.9·A647 + 8.08·A664.5.

### 4.5. Ion Determination

Plant material was lyophilized and finely ground for analysis. After calcination at 550 °C, cations were determined by inductively coupled plasma-optical emission spectrometry (ICP-OES) (Varian Vista-MPX, Varian Australia, Mulgrave, VIC, Australia).

### 4.6. Data Collection and Statistical Analysis

Five plants of each cultivar were used for this experiment. Samples were taken every 7 days: immediately after 30 days of the adaptation period (control), 7 days after waterlogging submission, and 7 days after a recovery phase following total drainage. Data were tested first for homogeneity of variance and normality of distribution. Significance was determined by analysis of variance (ANOVA), and the significance (*p* ≤ 0.05) of differences between mean values was tested by Duncan’s New Multiple Range Test, using Statgraphics Centurion^®^ XVI (StatPoint Technologies, Inc. Warrenton, VA, USA).

## 5. Conclusions

At the level of waterlogging used in this experiment, ‘Cleopatra’ was clearly the most affected genotype, showing changes in relative water content, stomatal conductance and, above all, photosynthesis. Many of these changes were also maintained during the recovery phase, and merit further study to ascertain the degree of irreversibility in these parameters. This did not occur in the other two rootstocks, since they showed signs of recovery in the case of those parameters that decreased during waterlogging. The results obtained in this study will contribute to a wider knowledge of the existing citrus germplasm and their responses to floods in a context where the capacity to resist these conditions will be key in the future of the Mediterranean basin citriculture.

## Figures and Tables

**Table 1:** Effects of waterlogging on nutrient composition in leaves of *Citrus macrophylla*, ‘Cleopatra’, and ‘Forner Alcaide no. 5′ citrus rootstocks after control, waterlogging, and recovery phases.

	Fe	B	Ca	Cu	K
*C. macrophylla*					
Phase					
Control	0.738 ± 0.101 ^a^	0.925 ± 0.085 ^b^	66.659 ± 6.454 ^a^	0.402 ± 0.051 ^a^	141.936 ± 9.805 ^a^
Waterlogging	0.789 ± 0.110 ^a^	1.213 ± 0.081 ^a^	67.069 ± 5.422 ^a^	0.284 ± 0.059 ^a^	124.163 ± 4.706 ^a^
Recovery	0.400 ± 0.059 ^b^	0.864 ± 0.034 ^b^	49.647 ± 3.897 ^b^	0.069 ± 0.017 ^b^	92.553 ± 3.999 ^b^
**Cleopatra**					
Phase					
Control	0.275 ± 0.039 ^a^	1.745 ± 0.265 ^a^	113.589 ± 14.323 ^a^	0.354 ± 0.051 ^a^	62.880 ± 9.963 ^a^
Waterlogging	0.273 ± 0.028 ^a^	1.640 ± 0.156 ^a^	100.264 ± 17.274 ^a^	0.237 ± 0.049 ^a^	50.248 ± 6.172 ^a^
Recovery	0.325 ± 0.047 ^a^	1.901 ± 0.208 ^a^	92.801 ± 18.480 ^a^	0.228 ± 0.097 ^a^	50.081 ± 6.231 ^a^
**Forner Alcaide no. 5**					
Phase					
Control	0.392 ± 0.046 ^a^	0.971 ± 0.061 ^b^	92.785 ± 3.341 ^a^	0.369 ± 0.045 ^a^	87.959 ± 1.158 ^a^
Waterlogging	0.461 ± 0.038 ^a^	1.338 ± 0.042 ^a^	95.149 ± 1.569 ^a^	0.349 ± 0.025 ^a^	84.107 ± 2.969 ^a^
Recovery	0.266 ± 0.017 ^b^	1.002 ± 0.070 ^b^	76.065 ± 3.768 ^b^	0.108 ± 0.028 ^b^	84.212 ± 1.239 ^a^
	**Mg**	**Mn**	**Na**	**P**	**Zn**
***C. macrophylla***					
Phase					
Control	0.963 ± 0.058 ^b^	0.186 ± 0.014 ^a^	18.605 ± 4.032 ^a^	10.061 ± 0.401 ^a^	0.128 ± 0.015 ^a^
Waterlogging	0.901 ± 0.057 ^b^	0.182 ± 0.019 ^a^	19.485 ± 5.260 ^a^	9.141 ± 0.494 ^a^	0.134 ± 0.009 ^a^
Recovery	1.630 ± 0.138 ^a^	0.100 ± 0.008 ^b^	14.190 ± 1.684 ^b^	9.959 ± 0.216 ^a^	0.080 ± 0.009 ^b^
**Cleopatra**					
Phase					
Control	3.067 ± 0.523 ^a^	0.085 ± 0.012 ^a^	39.952 ± 4.663 ^a^	11.884 ± 1.692 ^a^	0.133 ± 0.013 ^a^
Waterlogging	3.098 ± 0.232 ^a^	0.090 ± 0.001 ^a^	33.105 ± 3.493 ^a^	10.749 ± 0.862 ^a^	0.135 ± 0.003 ^a^
Recovery	2.459 ± 0.541 ^a^	0.096 ± 0.013 ^a^	27.156 ± 6.742 ^a^	9.645 ± 0.770 ^a^	0.119 ± 0.020 ^a^
**Forner Alcaide no. 5**					
Phase					
Control	2.867 ± 0.164 ^a^	0.102 ± 0.013 ^b^	9.467 ± 1.675 ^a^	13.401 ± 0.335 ^a^	0.153 ± 0.009 ^a^
Waterlogging	2.630 ± 0.152 ^a^	0.136 ± 0.008 ^a^	9.732 ± 0.446 ^a^	13.297 ± 0.273 ^a^	0.161 ± 0.006 ^a^
Recovery	2.790 ± 0.129 ^a^	0.087 ± 0.009 ^b^	9.957 ± 0.863 ^a^	11.029 ± 0.459 ^b^	0.106 ± 0.004 ^b^

Values are means ± SE. Different letters denote significant differences (*p* < 0.05) between phases in the same rootstock.

## Data Availability

Data will be available upon reasonable request.
